# Chemical and Structural Changes by Gold Addition Using Recharge Method in NiW/Al_2_O_3_-CeO_2_-TiO_2_ Nanomaterials

**DOI:** 10.3390/ma14195470

**Published:** 2021-09-22

**Authors:** Jorge Cortez-Elizalde, Ignacio Cuauhtémoc-López, Zenaida Guerra-Que, Alejandra Elvira Espinosa de los Monteros, Ma. Antonia Lunagómez-Rocha, Adib Abiu Silahua-Pavón, Juan Carlos Arévalo-Pérez, Adrián Cordero-García, Adrián Cervantes-Uribe, José Gilberto Torres-Torres

**Affiliations:** 1 Laboratorio de Nanomateriales Catalíticos Aplicados al Desarrollo de Fuen-tes de Energía y Remediación Ambiental, Centro de Investigación de Ciencia y Tecnología Aplicada de Tabasco (CICTAT), Universidad Juárez Autónoma de Tabasco, DACB, Km.1 Carretera Cunduacán-Jalpa de Méndez, Cun-duacán 86690, Tabasco, Mexico; LINK-190@hotmail.com (J.C.-E.); nachoftir@gmail.com (I.C.-L.); ale2962@gmail.com (A.E.E.d.l.M.); manlura9@hotmail.com (M.A.L.-R.); adibab45@gmail.com (A.A.S.-P.); carlos.arevalo@ujat.mx (J.C.A.-P.); adrian.cordero@ujat.mx (A.C.-G.); adrian.cervantes@ujat.mx (A.C.-U.); 2 Laboratorio de Investigación 1 Área de Nano-Tecnología, Tecnológico Nacional de México Campus Villahermosa, Km. 3.5 Carretera Villahermosa–Frontera, Cd. Industrial, Villahermosa 86010, Tabasco, Mexico; zenaida.gq@villahermosa.tecnm.mx

**Keywords:** gold addition, recharge method, NiW/Al_2_-O_3_-CeO_2_-TiO_2_ nanomaterials

## Abstract

NiWAu trimetallic nanoparticles (NPs) on the surface of support Al_2_O_3_-CeO_2_-TiO_2_ were synthesized by a three-step synthetic method in which Au NPs were incorporated into presynthesized NiW/Al_2_O_3_-CeO_2_-TiO_2_. The recharge method, also known as the redox method, was used to add 2.5 wt% gold. The Al_2_O_3_-CeO_2_-TiO_2_ support was made by a sol–gel method with two different compositions, and then two metals were simultaneously loaded (5 wt% nickel and 2.5 wt% tungsten) by two different methods, incipient wet impregnation and ultrasound impregnation method. In this paper, we study the effect of Au addition using the recharge method on NiW nanomaterials supported on mixed oxides on the physicochemical properties of synthesized nanomaterials. The prepared nanomaterials were characterized by scanning electron microscopy, BET specific surface area, X-ray diffraction, diffuse reflectance spectroscopy in the UV–visible range and temperature-programmed desorption of hydrogen. The experimental results showed that after loading of gold, the dispersion was higher (46% and 50%) with the trimetallic nanomaterials synthesized by incipient wet impregnation plus recharge method than with impregnation plus ultrasound recharge method, indicating a greater number of active trimetallic (NiWAu) sites in these materials. Small-sized Au from NiWAu/ACTU1 trimetallic nanostructures was enlarged for NiWAu/ACT1. The strong metal NPs–support interaction shown for the formation of NiAl_2_O_4_, Ni-W-O and Ni-Au-O species simultaneously present in the surface of trimetallic nanomaterial probably plays an important role in the degree of dispersion of the gold active phase.

## 1. Introduction

Supported bimetallic nanoparticles (NPs) alloys or even recently trimetallic NPs have been a strategy commonly used in nanomaterials reports [1,2,3,4,5,6,7,8]. It is well known that the nanostructured nanomaterials must be designed with high stability against leaching and agglomeration or sintering. 

The addition of second metal in the bimetallic system significantly enhances the stability and the activity of the designed catalysts due to a synergistic interaction [9]. Moreover, indeed, the addition of a third metallic component improves the stability of a second metal in a parent bimetallic system [6,10,11,12]. Jin et al. [10] found the enhanced stability of trimetallic alloy material (Ni-Fe-Cu) during dry reforming of methane (DRM) and weakened leaching of Fe. The Fe was affected by the reaction conditions of this application in the bimetallic system. 

The preparation methods can synthesize supported NPs in a single step or in two steps. In the case of a single step, both the precursor salt of the support and the active phase are added in the reaction mixture; otherwise, in sequential or two-step, first the support is synthesized, usually an oxide, and then the active phase, usually a metal, is prepared by some other specific method, expecting all the metal to be added and adsorbed on the support, without metal loss and with a high metal dispersion. The methods mentioned above for the nanostructure NPs depend on many factors such as the pH value, the calcination temperature, metal loading, nature of support and metal or metals, which have significant consequences on the catalytic properties of the NPs. These methods determine important properties such as homogeneous metal dispersion, high specific surface area, adequate acidity/basicity ratio, metal–support interaction, metal–metal interaction, metal–metal–metal interaction and generation of structural defects such as oxygen vacancies and reducibility [13,14,15,16,17]. 

Indeed, Mendoza-Nieto et al. [18] studied NiMoW trimetallic catalysts supported on SBA-15 and conventional γ-Al_2_O_3_ support in hydrodesulfurization (HDS), and they found an effect of used support on the catalytic behavior of HDS. They explained this effect by the strong metal–support (Al_2_O_3_) interaction due to the presence of a significant amount (75%) of Mo^6+^ and W^+6^ species in tetrahedral coordination and the weak interaction with the deposited metal species on SBA-15 due to the formation of agglomerated NiMoO_4_ and/or WO_3_ species in the catalyst and larger proportion of octahedrally coordinated metal species. Jahel et al. [19] demonstrated that the indium addition at higher loadings in trimetallic Pt/Al_2_O_3_SnIn–Cl naphtha-reforming catalyst decreases the acidity of the support and increases isomerization selectivity. Bocanegra et al. [20] synthesized InPtSn trimetallic NPs with different Sn contents supported on MgAl_2_O_4_. They concluded that trimetallic catalysts displayed a strong interaction between the different metals, which could be responsible for the good performance of these systems in catalytic dehydrogenation. The total acidity of the Pt–Ir-Ge trimetallic catalyst was slightly increased after Ge addition, as reported by Samoila et al. [21]. Liang et al. [22] synthesized highly dispersed non-noble trimetallic Cu-Ni-Co NPs supported on the pores of the metal–organic framework MIL-101, and they attributed the enhancement in the catalytic performance of the hydrolysis of ammonia borane to the large catalyst surface as well as the synergetic effect between trimetallic NPs. 

Gold in small particles (<5nm) supported on oxides could be active even at ambient temperature. The nanometric gold synthesized has shown a relationship between the kind of support that has been used and the catalytic activity of the nanometal [23,24,25]. In addition to the classic deposition–precipitation method (DP) proposed by Haruta et al. [26], different methods have been developed to prepare highly active Au catalysts [27,28,29,30,31,32,33,34,35,36]. Normally, these preparation methods can produce small gold particles (<10 nm) that are strongly linked to the support; most of them require total control of synthesis parameters due to the strong influence of the preparation conditions on the final characteristics of the material and therefore on its catalytic properties. 

A three-step synthetic method was selected for the present study. First, Al_2_O_3_-CeO_2_-TiO_2_ support was prepared by a sol–gel method with two different compositions, and then two metals were simultaneously loaded (5% nickel and 2.5% tungsten) by two different methods, incipient wet impregnation and ultrasound impregnation method. For the final step, for the formation of supported Au/NiW trimetallic NPs, we used the recharge method, also known as the redox method. The recharge method favors the deposit of the second metal on the first prereduced metal in order to create metal–metal interactions. A group of researchers from the University of Poitiers led by Professor Barbier [37] named it the redox method or recharge method, in which surface reactions modify the catalyst between chemisorbed hydrogen over the first metal and the cation of the second metal according to the following scheme:

$$nH_{\mathrm{ads}}+Mn^{+}\;\to M_{\mathrm{ads}}+n\;H^{+}$$

where H_ads_ is the adsorbed hydrogen over the metal surface, Mn**^+^** is the cation of the second metal in solution and M_ads_ is the second adsorbed metal.

The preparation strategy used in this study was developed in order to generate small trimetallic NPs and to favor strong metal–support interaction and strong metal–metal–metal interaction. The aim of this work was to disperse gold NPs below 5 nm into the mixed oxide Al_2_O_3_-CeO_2_-TiO_2_ prepared by sol–gel method modified with Ni and W to study the effect that Au nanoparticles have on the physicochemical properties of these trimetallic NPs materials due to intimate contact between three metal constituents in the catalyst nanostructure.

## 2. Materials and Methods

### 2.1. Materials Preparation

The Al_2_O_3_-CeO_2_-TiO_2_ supports were prepared via the sol–gel method; these were prepared varying weight percentages of aluminum, cerium and titanium oxide (90 wt% Al_2_O_3_, 1 wt% CeO_2_ and 9 wt% TiO_2_ named ACT1 and 94 wt% Al_2_O_3_, 1 wt% CeO_2_ and 5 wt% TiO_2_ named ACT2). The metallic precursors used were aluminum tri-sec-butoxide Al[OCH(CH_3_)C_2_H_5_]_3_ (97% Aldrich), titanium butoxide (IV) Ti[O(CH_2_)_3_CH_3_]_4_ (97% Aldrich), cerium nitrate (III) hexahydrate Ce(NO_3_)_3_ (99.999% Aldrich) and a mixture of water and n-butanol (99.9%, Baker), in relation to alkoxide/butanol 1:8 in volume and alkoxide/water 1:16 in volume. They were aged at 70 °C for 24 h and dried in a rotary evaporator; finally, the samples were calcined at 550 °C for 12 h using a heating ramp of 2 °C/min. 

The four bimetallic supported catalysts were obtained by the wet impregnation method and the ultrasound method. The incorporation of the active metal phase on Al_2_O_3_-CeO_2_-TiO_2_ support was conducted to obtain a nominal 5 wt% Ni and 2.5 wt% W loading.

Deposition of Ni and W into the modified supports was carried out by the wet impregnation method according to the following procedure: First, 5 wt% Ni and 2.5 wt% W were loaded in 10 g of support, and 100 mL of the precursor salt of Ni(NO_3_)_2_.6H_2_O (Sigma-Aldrich) and hydrated ammonium metatungstate (NH_4_)6H_2_W_12_O_40_XH_2_O (85%, Aldrich Chemistry) was used to synthesize the active phases and metal promoters. They were subjected to a calcination process at 2 °C/min in airflow/O_2_ at 400 °C; subsequently, they were reduced in H_2_ flow at 400 °C for 4 h. 

The ultrasound method was used to impregnate 5 wt% Ni and 2.5 wt% W in 5 g of support in 50 mL of water under vibration in ultrasound (8890-Cole Parmer); the prepared materials were dried in a rotary evaporator and then dried in an oven at 120 °C for 12 h. Afterward, they were calcined using a heating ramp of 2 °C/min in airflow/O_2_ at 400 °C for 4 h. Finally, they were reduced in H_2_ flow at 400 °C for 4 h using the same heating ramp as for the calcination.

**Figure 1:**
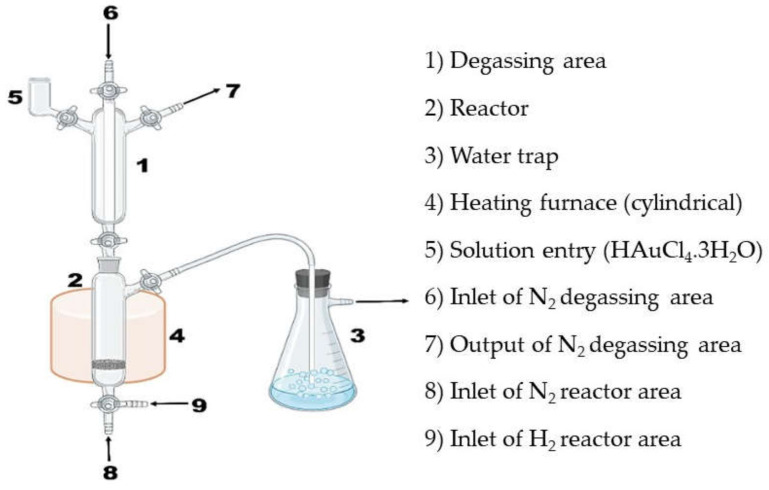
Synthesis system for the preparation of NiWAu/Al_2_O_3_-CeO_2_-TiO_2_ nanomaterials by recharge method.

The recharge method was used to impregnate the supported bimetallic Ni-W NPs with 2.5 wt% Au, applying hydrated tetrachloroauric acid as a precursor (HAuCl_4_.3H_2_O). The amount of bimetallic NPs needed to prepare 2 g of trimetallic NPs was introduced into the quartz reactor. First, the NiW/Al_2_O_3_-CeO_2_-TiO_2_ bimetallic catalyst was reduced with hydrogen at a temperature of 400 °C, and then 5 mL of the solution HCl was added at 0.2 M. Then, a gold solution HAuCl_4_.3H_2_O was introduced at 2.5 wt%. Finally, the nanocatalysts were activated by reduction of hydrogen at a temperature of 400 °C for 4 h using the same heating ramp of the above-mentioned heat treatments. Figure 1 shows the system used to prepare the materials by the recharge method. The Table 1 shows the materials prepared, their compositions, their metal loads, the preparation method and the code assigned.

### 2.2. Materials Characterization

#### 2.2.1. X-ray Diffraction

The equipment used was a Bruker AXS model D8 Advance diffractometer (Borken, North Rhine-Westphalia, Germany). A Cu anode was used; the radiation corresponded to the transition CuKα with a wavelength of 1.5418 Å from 20 to 80° in the scale of 2θ, with a step size of 0.02° and a time per step of 1 s.

#### 2.2.2. UV-Vis Diffuse Reflectance Spectroscopy (UV-Vis DRS)

The diffuse reflectance spectra of the synthesized nanomaterials were obtained with a UV-Vis Varian Cary 300 spectrophotometer (Varian Inc., Palo Alto, CA, USA), provided with an integration sphere, useful for the powder analysis. The 190–800 nm region was analyzed using BaSO_4_ as a white reflectance standard to obtain the baseline.

#### 2.2.3. BET Specific Surface Area (SSA)

The textural property characterization of the supports and bimetallic and trimetallic nanomaterials was carried out by physical adsorption of N_2_ (Praxair 5.0 U.A.P.) at −198 °C using a Micromeritics Model TriStar II (Micromeritics Instrument Corporation 4356 Communications Drive, Norcross, GA, USA). BET specific surface area determinations were performed using the Brunauer, Emmett and Teller (BET) method; pore volume (Vp) and pore size distribution (PSD) were assessed by the BJH method. Prior to nitrogen adsorption analysis, the samples were degassed under nitrogen flow at 150 °C overnight.

#### 2.2.4. Scanning Electron Microscopy (SEM)

Energy dispersive X-ray spectroscopy (EDX) coupled with scanning electron microscopy (SEM) was used to appreciate the morphology, and semiquantitative analysis was performed with the BES detector of particles corresponding to the materials synthesized in a JEOL brand model JSM-6010LA (Jeol Ltd, Akishima Tokyo, Japan).

#### 2.2.5. Transmission Electron Microscopy (TEM)

Transmission electron microscopy (TEM) was performed with a JEOL JEM2100 STEM (Jeol Ltd, Akishima Tokyo, Japan). The samples were ground, suspended in ethanol at room temperature and dispersed with agitation in an ultrasonic bath for 15 min; then, an aliquot of the solution was passed through a carbon copper grid. The particle size distribution of the catalysts was obtained by measuring more than 200 nanoparticles in each sample.

The particle average diameter (dm) was calculated using the formula:

(1) $$dm=\sum_{i}(x_{i}d_{i})/\sum_{i}x_{i}$$

where $x_{i}\;$is the number of particles with diameter $d_{i}$.

#### 2.2.6. Temperature-Programmed Desorption of Hydrogen (TPD-H_2_) 

This characterization technique used an automated chemisorption analyzer, model Belcat B (Bel-Japan) with thermal conductivity detector, using 0.2 g of catalyst. First, the samples were pretreated with the following protocol: 20% O_2_/H_2_ for 30 min at 400 °C, 20% O_2_/H_2_ for 1 min at 35 °C, He for 60 min at 35 °C, 5% H_2_/Ar for 30 min at 400 °C, 5% H_2_/Ar for 1 min at 35 °C with a flow rate of 50 sccm. Then, the samples were treated with Ar at 50 sccm. The temperature was raised from room temperature to 400 °C at a heating rate of 10 °C min^−1^. Dispersion was calculated according to mmol g^−1^ of H_2_ adsorbed on each sample, metal content of nickel (5 wt%) and 1:1 Ni:H stoichiometry, metal content of tungsten (2.5 wt%) and 1:1 W:H stoichiometry and metal content of gold (2.5 wt%) and 1:1 Au:H stoichiometry.

## 3. Results and Discussion

### 3.1. Materials Characterization 

#### 3.1.1. BET Specific Surface Area (SSA)

The nitrogen physisorption results of the supports and the supported bimetallic and trimetallic NPs catalysts are listed in Table 2. The supported NiWAu trimetallic NPs are mesoporous amorphous solids with high SSA in the range of 220 to 215 m^2^ g^−1^ for the catalysts impregnated by wet impregnation plus recharge method; on the other hand, for the trimetallic catalysts impregnated by ultrasound method plus recharge method, the SSA was increased in the range of 280 to 290 m^2^ g^−1^. Another important observation deals with the increasing SSA of the four supported NiWAu trimetallic NPs with respect to the four supported NiW bimetallic NPs, indicating that the recharge method plays an important role in the support and metal redispersion [36]. 

**Figure 2:**
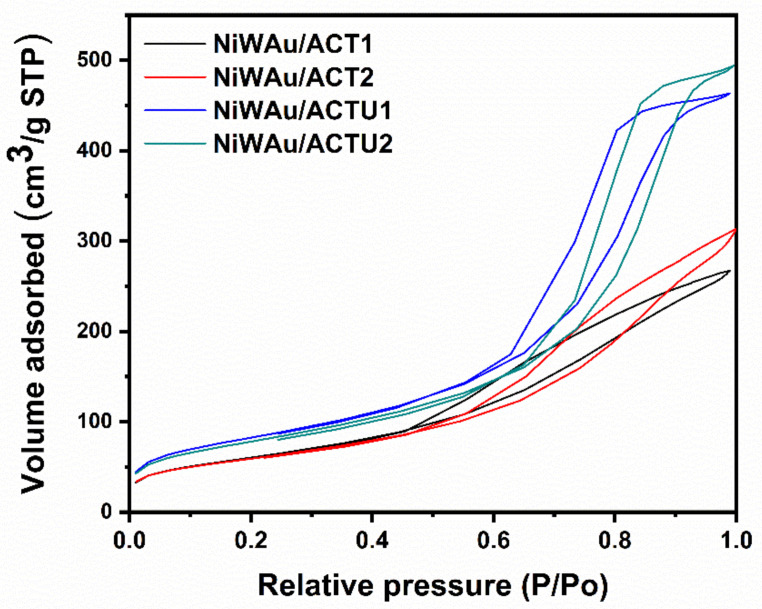
BET adsorption–desorption isotherms for the four supported trimetallic NPs nanomaterials.

**Figure 3:**
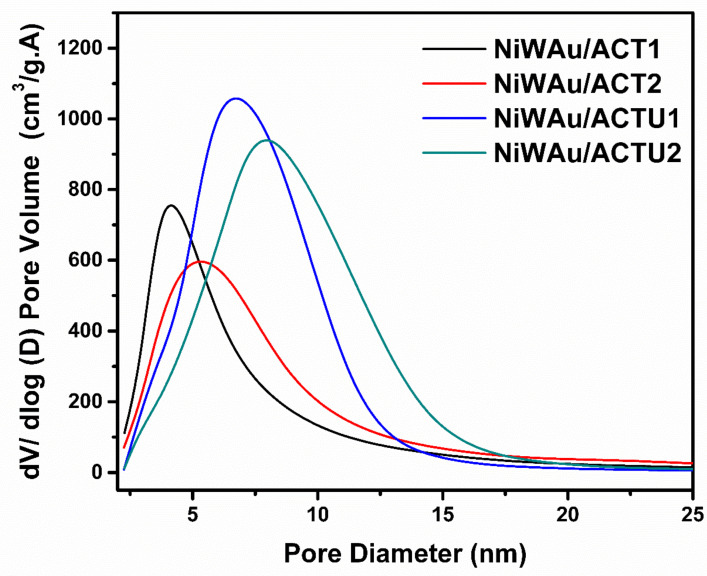
Pore distribution of the four supported trimetallic NPs nanomaterials synthesized by the recharge method.

Figure 2 shows the adsorption–desorption isotherms of the prepared catalysts, showing type IV isotherms according to the IUPAC, which are characteristic of a mesoporous material ranging from 2 to 50 nm, with a hysteresis curve type H1 characteristic of the geometry of tubular shaped capillaries opened at the ends and capillaries shaped like an ink bottle. In Figure 3, it can be observed that the average pore diameters ranged from 8 to 14 nm.

#### 3.1.2. X-ray Diffraction (XRD)

**Figure 4:**
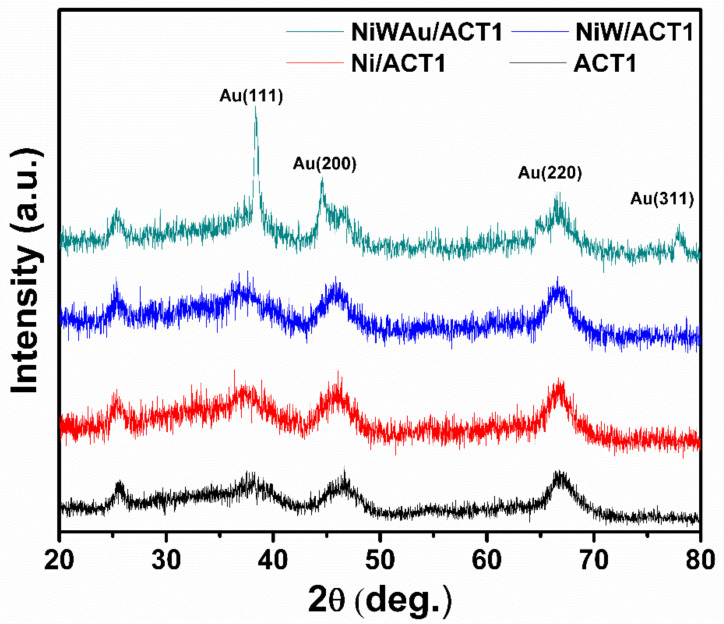
XRD patterns of NiWAu/ACT1 nanomaterials and support.

Figure 4 displays the XRD patterns of NiW/ACT1 and NiWAu/ACT1 fresh nanomaterial samples. The diffraction patterns of prepared support and bimetallic and trimetallic NPs supported on ACT1 can be seen. After the thermal treatments of reduction, it was observed that the NiW/ACT1 and NiWAu/ACT1 fresh samples preserved the crystalline phases of gamma-alumina and the anatase phase of titania. The diffraction peak at 37.65° indicates the formation of very small NiO crystallites [38,39]. Figure 4 also shows the diffraction patterns of Au in NiWAu/ACT1, where peaks at 38.30, 44.48, 64.82 and 77.77° correspond to the (111), (200), (220) and (311) crystalline planes of gold. Representing a face-centered cubic (FCC) structure of the gold NPs on the support surface, gold is added in a metallic form due to the reduction by hydrogen adsorbed in nickel and tungsten [8,29]. An oxide-reduction process occurs, causing metal–metal–metal interaction. 

**Figure 5:**
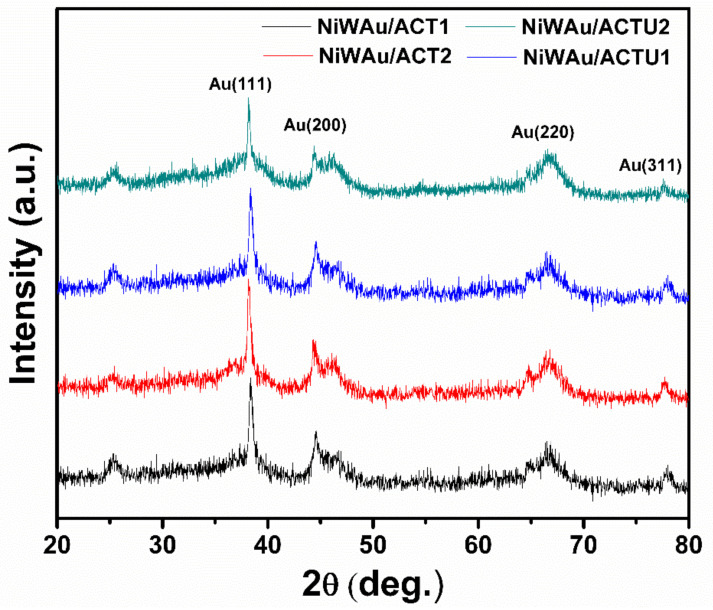
XRD patterns of NiWAu/ACT and NiWAu/ACTU nanomaterials.

Figure 5 shows the X-ray diffraction patterns of the deposited gold catalysts in NiWAu/ACT1, NiWAu/ACT2, NiWAu/ACTU1 and NiWAu/ACTU2 that are used to identify crystalline phases of the nanostructured materials. For all samples, the diffraction peaks were observed for the Au nanoparticles at 38.30, 44.48, 64.82 and 77.77°. The average particle size of the four supported trimetallic NPs was estimated using the Scherrer equation, providing an average particle size of gold smaller than 5 nm (see Table 3). The increase in the intensity of the peaks at 46.44 and 66.75° is due to the formation of NiAl_2_O_4_ nickel aluminate in the four trimetallic nanomaterials; this is confirmed by UV-Vis DRS, which shows the NiAl_2_O_4_ formation, i.e., a spinel phase nanostructure [39].

#### 3.1.3. UV-Vis with Diffuse Reflectance of Solids (UV-Vis DRS)

**Figure 6:**
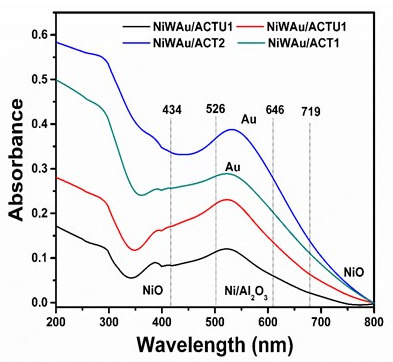
UV-Vis with diffuse reflectance of solids (DRS) for nanomaterials synthesized by the recharge method.

Figure 6 shows the results of UV-Vis reflectance spectroscopy for nanomaterials with gold. The TiO_2_ bands are related to the charge transfer of O^2--^^→^Ti^4+^; the presence of crystalline CeO_2_ absorbs strongly in the UV region close to 400 nm [40]. There is the presence of absorption bands at wavelengths below 350 nm, and this indicates the existence of fine crystallites, not detectable by XRD. The bands at 526 and 634 nm are assigned to Ni^2+^ ions in the tetrahedral symmetry in the lattice of Al_2_O_3_, and they are associated with the formation of the spinel phase Ni_2_O_4_ also detected by XRD [41]. The UV-Vis DRS studies suggest the formation of surface Ni/Al_2_O_3_ across the bands at 598 and 636 nm. The peaks at 462 and 710 nm are characteristic of NiO crystallites formation. The band at 710 nm is associated with octahedral Ni^2+^ symmetry and presents low intensity. These latter interactions of Ni/Al_2_O_3_ and the formation of NiO crystals are also seen in XRD [39,42].

Absorption bands were shown at 300 to 350 nm corresponding to W^6+^ species and Ni-W–support interactions. W^6+^ and WOx species in tetrahedral coordination, with octahedral symmetry Ni ions, Ni [Ni^2+^6O^−2^], and WOx species in octahedral coordination with Ni ions [Ni^2+^4O^−2^] with tetrahedral symmetry were also shown. 

The position of the Au band in the metallic state is generally accepted as between 500 and 600 nm (plasmon band). The band position of the Au species is undefined; however, it has been reported that the Au^+^ cations exhibit an absorption band at around 240 nm, while small clusters such as (Au)_n_^δ+^ exhibit a band at around 390 nm [43]. A band at 525 nm is shown, which is typical of the Au plasmon, because of small metallic Au particles in the trimetallic catalysts, which confirms the presence of Au^0^. The type of absorption band is due to the partial loading of gold nanoparticles. A higher peak in XRD/UV-Vis implies a larger particle size [44,45].

#### 3.1.4. Scanning Electron Microscopy (SEM) 

**Figure 7:**
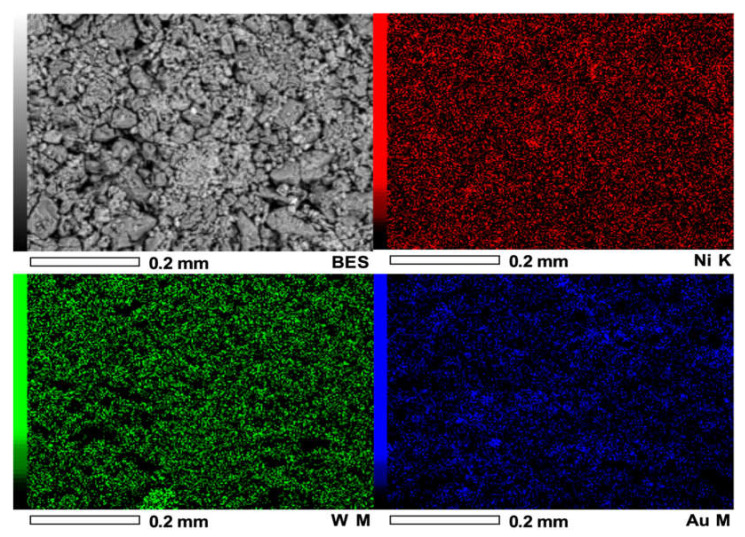
EDX elemental chemical mapping of NiWAu/ACT1 nanomaterial.

Figure 7 shows the SEM image and the energy-dispersive spectrometry (EDS) elemental mapping images of a selected region of NiWAu/ACT1. The analysis of Ni, W and Au elemental mapping suggested that the trimetallic NPs are homogeneously distributed over the entire support surface and showed heterogeneous morphology of the support ACT1 consisting of crystals of different sizes, shapes and orientations.

The elemental composition of the NiWAu trimetallic NPs supported on Al_2_O_3_-CeO_2_-TiO_2_ synthesized by two different routes is reported in Table 4. It highlights a fact concerning the final total loading of Ni and Au in the trimetallic NPs. The impregnating solutions were adjusted to reach a nominal Au loading of 2.5 wt% and Ni loading of 5 wt%. However, lower values of Au loading of 2.5 wt% and Ni loading of 5 wt% were obtained with the preparation route that was selected by ultrasound plus recharge method, which is opposite to that observed in the case of supported trimetallic NPs prepared by wet impregnation plus recharge method. Therefore, the Au loading of the NiWAu/ACT1 was very close to the target gold content, indicating the enhanced resistance against the loss of the Au precursor after the synthetic method of trimetallic NPs. This latter result shows the effect of the preparation methods, and it could be related to a stronger interaction of the support with the NiWAu trimetallic NPs. The presence of NiAl_2_O_4_ nickel aluminate and Ni-W-O is evidence of a close intimate interaction between metals and support that indeed promotes their redox properties.

**Figure 8:**
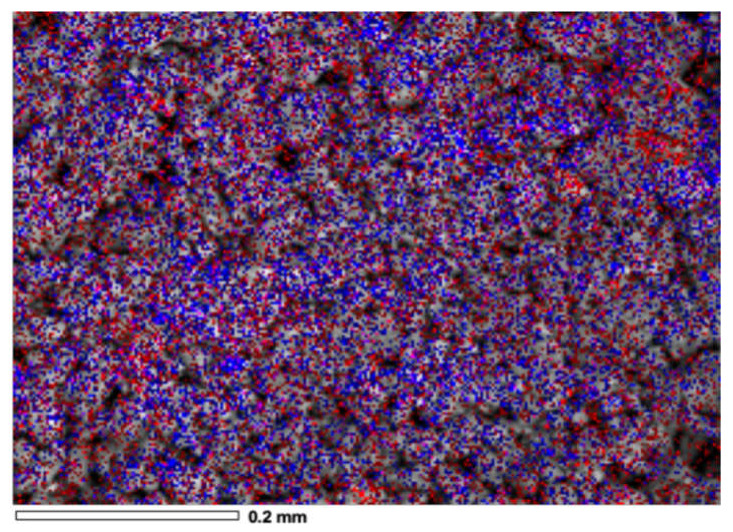
Overlapping EDX elemental mapping of NiWAu/ACT1 nanomaterial with gold at 2.5 wt%.

**Figure 9:**
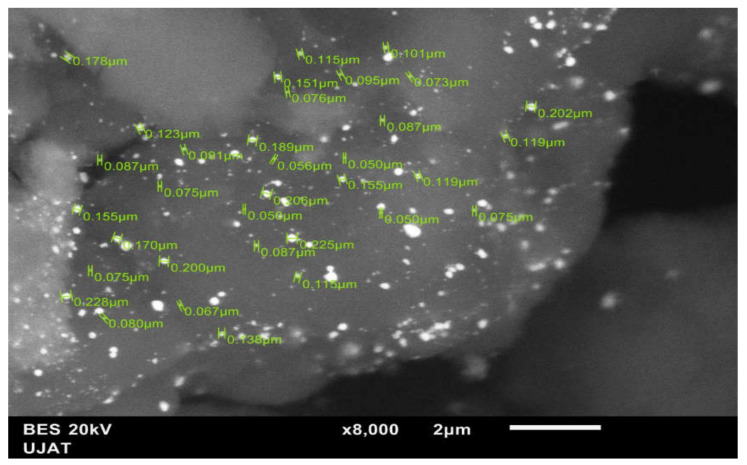
SEM images with BES detector for the nanomaterial NiWAu/ACT1.

**Figure 10:**
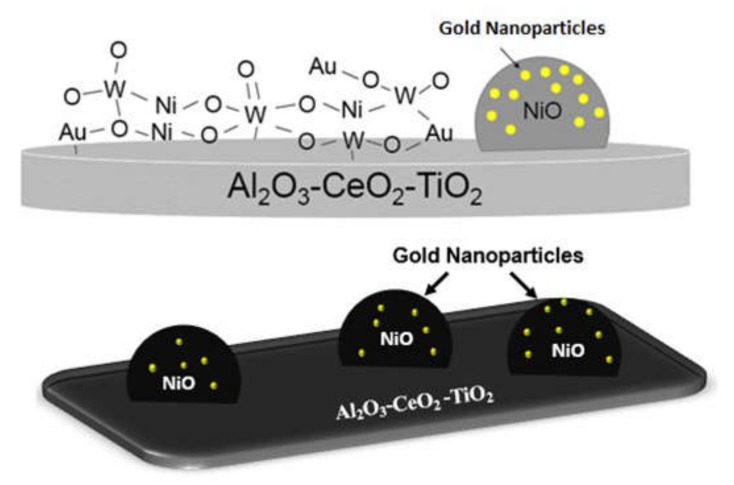
Nanoparticles of Ni and Au distributed on the support for NiWAu/ACT1 nanomaterial.

Figure 8 displays the mutual overlap of three EDX elemental maps, Ni, Au and W. From the EDX image, it was indicated that Au and Ni elements were intermixed, showing gold NPs predominant with a good dispersion on the Ni surface near the support lattice. It can be concluded that the nickel NPs are incorporated in the gold matrix due to the metallic interaction between nickel and gold [46,47,48]. Metal–support interactions in the supported catalysts have been detected when the incorporation of metals into the framework of a support lattice occurs [49,50]. In this work, the metal–metal–support interaction (Ni-W-O) and NiAl_2_O_4_ also could be seen in the UV-Vis DRS results for the four trimetallic supported NPs nanomaterials. In fact, the recharge method influenced the redistribution of the metals in the boundary of the support, which probably provoked a redispersion effect of the metals on the catalyst surface and the intimate contact between metals and support. The morphological micrograph observed in Figure 9 is for Au/NiW/Al_2_O_3_-CeO_2_-TiO_2_ synthesized by the recharge method. By correlating data from DRX and UV-Vis DRS, Figure 10 shows the deposit of the nanometer gold around the NiO crystallites. 

#### 3.1.5. Transmission Electron Microscopy (TEM)

**Figure 11:**
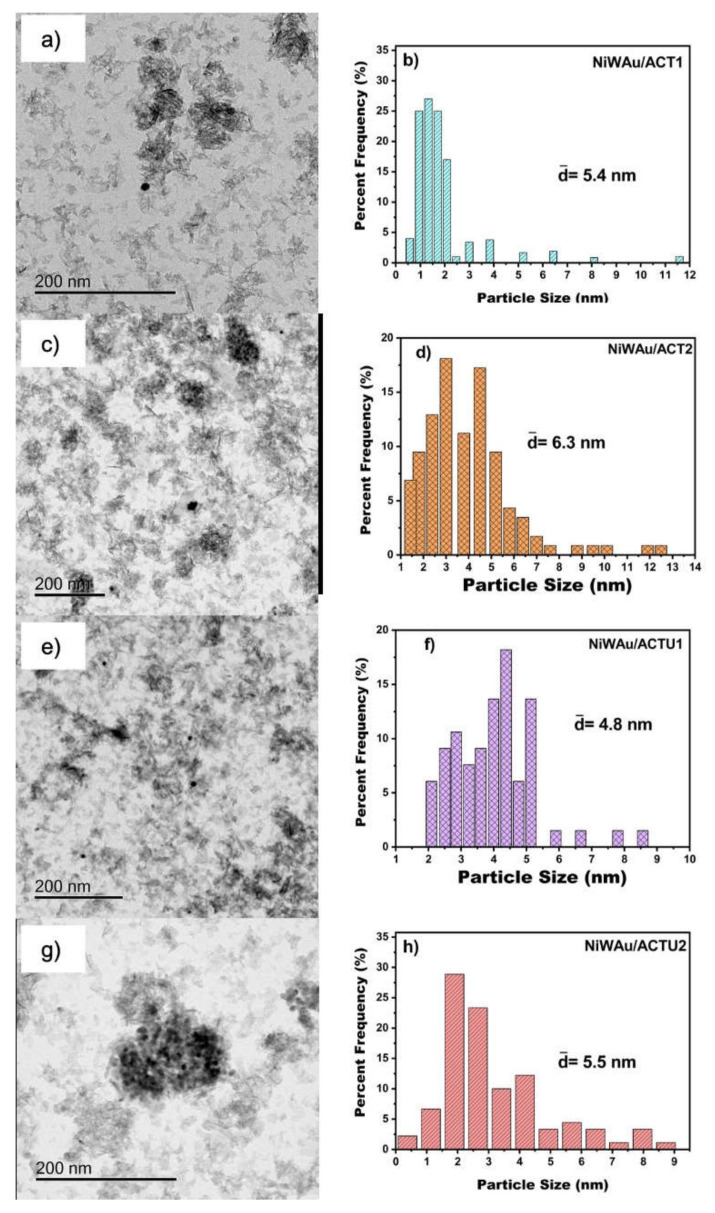
TEM image and particle size distributions (right side) of the nanomaterials (**a**,**b**) NiWAu/ACT1, (**c**,**d**) NiWAu/ACT2, (**e**,**f**) NiWAu/ACTU1 and (**g**,**h**) NiWAu/ACTU2 prepared by the recharge method.

Representative TEM images and particle size distributions of fresh supported trimetallic NPs NiWAu/ACT1, NiWAu/ACTU1, NiWAu/ACT2 and NiWAu/ACTU2 can be appreciated in Figure 11. For each sample, about 200 individual particles randomly selected in a unique zone of the nanomaterial were analyzed. The metal nanoparticles of gold, nickel and tungsten appeared darker in the images because they showed strong electron diffraction. In the two samples, the shape of the particles mostly is spherical or quasispherical, although a few particles represented cylindrical shape. The TEM image of supported NiWAu trimetallic NPs showed several agglomerates and many separated or isolated spherical trimetallic NPs distributed over the whole surface [44,48]. Analysis of the particle size distributions shows a medium distribution with most of the particle sizes ranging between 1 and 12 nm. The average particle size of the trimetallic NPs were 5.4, 6.3, 4.8 and 5.5 nm for NiWAu/ACT1, NiWAu/ACT2, NiWAu/ACTU1 and NiWAu/ACTU1, respectively. Similar values of the particle size were obtained from the XRD line width (see Table 3).

#### 3.1.6. Temperature-Programmed Desorption of Hydrogen (TPD-H_2_) 

The accessibility of nickel, tungsten and gold was determined from the thermogram areas of the TPD-H_2_, assuming a stoichiometry of H/Ni=1, H/W=1 and H/Au=1 taking into account the values of Table 5 for calculations. 

**Figure 12:**
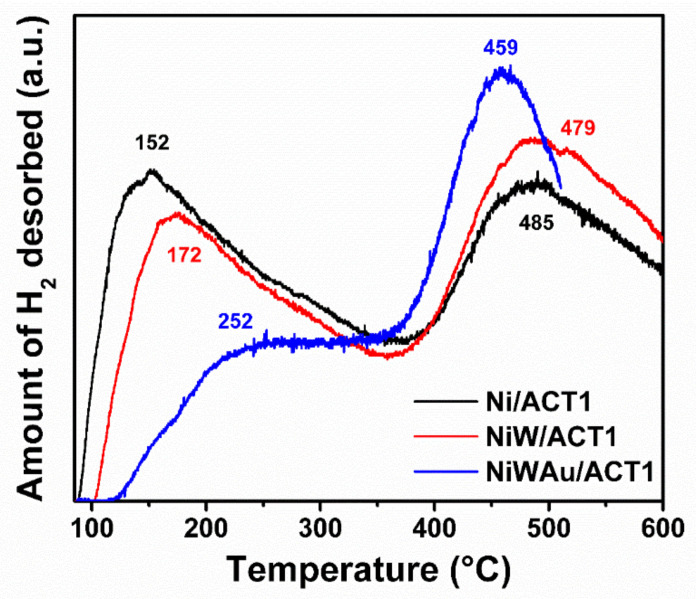
TPD-H_2_ profiles of reduced trimetallic nanomaterials at 400 °C.

These thermograms of TPD-H_2_ show the peaks originating from the desorption of hydrogen. The area under the curve of Figure 12 represents the desorbed moles of hydrogen in the material, and therefore, each peak represents a desorption temperature. Additionally, the TPD-H_2_ method was used to define the types of catalytic active sites for hydrogen chemisorption and activation; furthermore, it was used to determine the influence that gold addition might have on the nature of catalytic active sites. 

Figure 12 shows the TPD-H_2_ profiles of supported NiW bimetallic NPs prepared by two different sequential methods (ultrasound and wet impregnation) and Au/NiW trimetallic NPs prepared after gold addition over the supported NiW bimetallic NPs. All the nanomaterials studied had two characteristic peaks in their TCD signal profile. Furthermore, the TPD-H_2_ profiles have two domains of H_2_ desorption peaks. The first domain includes desorption peaks at lower temperatures, at around 150–250 °C. The second domain is situated at higher temperatures, at around 450–490 °C. The first domain is largely recognized to represent hydrogen desorbed from metallic nanoparticles. The second one can be ascribed to hydrogen originally located on subsurface layers and/or to spillover hydrogen [51,52]. The H_2_ spillover occurs during the prereduction step of TPD-H_2_ experiments, when the hydrogen atoms are dissociated over the nanometallic surface and migrate to subsurface layers and/or to the support, generating hydrogen species strongly bonded to the nanomaterial surface. The peaks related to exposed trimetallic NPs and active metal sites are located at lower temperatures [21,51]. 

The metal accessibility and the particle size present in the supported NiW bimetallic and AuNiW trimetallic NPs were determined using the values of the Table 5 (metal surface and densities) and the results obtained by TPD-H_2_ profiles shown in Table 6. It was observed that when gold was added to the NiW bimetallic nanomaterials synthesized by the impregnation method, the dispersion of metallic surface species improved significantly, therefore increasing the exposed trimetallic surface %D.

The results of nitrogen physisorption showed values of high specific areas that increased after the addition of Au by the recharge method due to the redispersion of the metals and the support in the catalyst, allowing the migration of Ni and W atoms. Moreover, there was an increase in metal dispersion or accessibility (%D) obtained by TPD-H_2_ where the available metallic surfaces increased when gold was added due to the effect of the recharge method, resulting in metallic crystallite average size values of around 1.7 to 2.1 nm, which correspond to well-dispersed gold, nickel and tungsten nanoparticles in the synthesized trimetallic nanomaterials.

One advantage of the wet impregnation method is that there is no loss of material on the first and second metals (Ni and W) deposited over the support; this causes a synergy to deposit the proposed theoretical Au content. On the contrary, for the ultrasound method, there was a loss of more than one-half of nickel and one-half of gold impregnated. Furthermore, the deposit of the nanometric gold is limited by the amount of the first two metals in the support. The average trimetallic (gold, nickel and tungsten) particle size measured though TPD-H_2_ was smaller when impregnation and recharge methods were selected for preparation.

#### 3.1.7. Structural and Catalytic Properties of Nanomaterials

The trimetallic supported nanocatalysts developed and synthesized in this study possess particular structural properties allowing them to be used for several catalytic applications. The close intimate interaction between metals and support (strong metal–support interaction) is a highly desirable structural property for nanocatalysts as it promotes their redox properties [53,54]. The strong metal–support interaction has a relationship with the number of oxygen vacancies in the support [54,55,56]. CeO_2_ has attracted much interest in the oxidation process due to its high oxygen storage capacity and unique redox ability. Its ability to store and release oxygen due to the effective redox Ce^4+^/Ce^3+^ sites that enable the exchange of oxygen via oxygen vacancy significantly increases the performance of catalytic systems and suppresses the deactivation of catalysts under rigorous reaction conditions [57,58]. For the degradation of refractory organic compounds (ROCs), it has been reported that the oxygen storage capacity (OSC) and the redox properties of ceria should be increased by the introduction of other transition and nontransition metal ions; therefore, many ceria-based catalysts have been developed, such as CeO_2_-ZrO_2_ [57,58,59,60,61], CeO_2_-TiO_2_ [62,63,64], CeO_2_-WO_3_ [65], CeO_2_/Al_2_O_3_ [66,67] and CeO_2_-SiO_2_ [68,69,70]. Besides, the species Ti^3+^ for the TiO_2_ support is related to oxygen vacancies as a result of lattice distortion or surface defects [71]. 

This oxygen vacancy is beneficial for forming reactive centers or yielding active oxygen, especially beneficial for oxidation degradation. They are acid sites called Lewis sites where a nucleophilic substrate can be deposited [72]. Previous works have proved that the number of acid sites promotes efficient catalytic properties (e.g., in catalytic wet air oxidation of ROCs [37,54,73,74,75,76] and production of biofuels [77,78,79]). 

The catalytic activity of degradation of ROCs via CWAO using heterogeneous nanocatalysts has been improved to increase the OSC directly associated with oxygen vacancies. The oxidation mechanism proposed by our group shows that such vacancies are directly involved in the oxygen activation reaction at the catalyst surface and, consequently, the creation of highly reactive surface oxygen species such as superoxides and peroxides [54,80,81,82,83]. The oxidation of ROCs can start by activating the oxygen molecule or the ROC, and oxygen may participate in the reaction as an adsorbed species on the catalyst surface. The Lewis acid sites could activate the electronic doublet of oxygen [53,73,74]. In any case, the presence of a nanocatalyst creates an ionic environment that favors the heterolytic reactions. In the case of aromatic compounds such as phenol, the ring-opening reaction can be produced either by a free redox radical mechanism (hemolytic rupture) or by an ionic (heterolytic) mechanism [73]. 

The mineralization process can be explained by the transformation of the aromatic compounds into aliphatic compounds by the ring-opening reactions. The aromatic molecule ring is oxidized to catechol, hydroquinone and benzoquinones (intermediates). Successively, the ring breaks into carboxylic organic acids of low molecular weight (<C6) such as carboxylic acids (maleic acid, acetic acid, formic acid, oxalic acid). In other words, the catalytic oxidation degradation pathway of phenol includes the hydroxylation (hydroquinone, catechol, o-benzoquinone) and organic acids until preferably producing CO_2_ and H_2_O [84,85,86]. When the mechanism includes the formation of oxidized C_6_-aromatic of hydroquinone and catechol, the occurrence of both compounds indicates parallel reaction pathways [87]. The presence of catechol and hydroquinone may be attributed to hydroxyl radical attack at ortho and para positions of the aromatic ring due to the resonance effect of phenol. Apart from the above reactions, a solid residue may be also formed as a result of the combination of phenyl radical with hydroquinone and p-benzoquinone through a series of chain reactions [88]. Acetic acid is especially accumulated in the system, which could be considered as the final product. If the mineralization process reaches complete total oxidation, the conversion of organic molecules to CO_2_ and H_2_O occurs. The TOC yield removal will be 100% of CO_2_. Therefore, the TOC removal parameter is directly associated with the selectivity of CO_2_. However, partial oxidation commonly occurs, and this causes the simultaneous presence of intermediates and CO_2_ in the reaction mixture. Nevertheless, the increase in TOC removal means that the organic refractory molecule will be more oxidized to CO_2_ [74,75,76].

It was found by our group that CeO_2_, due to its ability to store and release oxygen and the effective redox Ce^4+^/Ce^3+^ occurring in its oxygen vacancies, plays an important role in enhancing the CO_2_ selectivity as explained by several authors. The formation of Ce^4+^-$O_{2}^{-}$–M at the interface could favor oxygen transfer between the nanocatalyst surface and the adsorbed species by a redox mechanism. It is important to highlight with our findings that the excess of Ce loading (50 wt%) increases OSC, which favors the para-oxidation of phenol and the consequent occurrence of carbon deposit by the polymer formation from p-benzoquinone and the decrease in the number of Lewis sites. It was established by our group that the number of total sites increases for the monometallic (Ru/ZrO_2_-CeO_2_) and bimetallic (RuAu/ZrO_2_-CeO_2_) catalysts by Ce loading of 10 wt%, and this enhances the ortho-oxidation of phenol. On the contrary, the number of total sites decreases when the Ce loading reaches 20 wt% in RuAu/ZrO_2_-CeO_2_ catalysts [53]. Another important contribution by our group is the finding that if the route takes place by the formation of the quinones, the polymerization on the surface of the catalysts is important; therefore, the deactivation of the material can be carried out due to the blocking of the superficial active sites [74,88].

Furthermore, another important application that has been discussed by our group is the production of biofuels through biomass. The conversion of glucose to produce 5-hydroxymethylfurfural (HMF) using TiO_2_–ZrO_2_ binary oxides and Al_2_O_3_-TiO_2_-W has been researched. The number of acid sites plays an important role in achieving the highest 5-HMF yield [76,77,78,79].

## 4. Conclusions

Four NiWAu supported trimetallic NPS nanomaterials were prepared by a three-step synthetic method in which the gold addition was the last step via the recharge method. The effect of the gold addition using the recharge method on the structural and chemical properties of NiW/Al_2_O_3_-CeO_2_-TiO_2_ was investigated. The TPD-H_2_ results obtained revealed that the addition of gold synthesized by impregnation plus recharge method improved the dispersion of trimetallic surface species (46% and 50%). The particle size of gold from XRD and TEM data in NiWAu/ACTU1 was smaller using impregnation plus ultrasound recharge method than impregnation plus recharge in NiWAu/ACT1.

The recharge method promoted nanostructures of gold nanoparticles <6.5 nm in the four NiWAu trimetallic catalysts supported on mixed oxides. After the addition of gold in the four supported bimetallic NiW systems, the specific surface areas (SSAs) were larger than those of the bimetallic systems. This method provided the rearrangement of the metallic surface, which provoked a redispersion effect of the metals on the catalyst surface. Gold is added in its metallic form due to the reduction produced by the adsorbed hydrogen in the supported Ni-W bimetallic NPs surfaces. 

The gold addition for the supported NiW/ACT1, NiW/ACT2, NiW/ACTU1 and NiW/ACTU2 bimetallic NPs enhanced the formation of NiAl_2_O_4_ nickel aluminate, Ni-W-O and Ni-Au-O phases, which were shown to be strong metal–support interaction species that have close intimate interaction between metals and support. These changes could not be seen in the four bimetallic systems.

## Figures and Tables

**Table 1:** Nanomaterials synthesized by the wet impregnation, ultrasound and recharge method.

Method	Weight Percentage		Material	Code
	Nickel (Ni)	Tungsten (W)		
Wet impregnation	5%	2.5%	NiW/Al_2_O_3_-CeO_2_ –TiO_2_ 90% 1% 9%	NiW/ACT1
			NiW/Al_2_O_3_-CeO_2_ –TiO_2_ 94% 1% 5%	NiW/ACT2
Ultrasound impregnation	5%	2.5%	NiW/Al_2_O_3_-CeO_2_ –TiO_2_ 90% 1% 9%	NiW/ACTU1
			NiW/Al_2_O_3_-CeO_2_ –TiO_2_ 94% 1% 5%	NiW/ACTU2
Recharge	Gold (Au)	2.5%	NiWAu/ACT1	NiWAu/ACTU1
			NiWAu/ACT2	NiWAu/ACTU2

**Table 2:** BET specific surface area (SSA) of the supports and metallic nanomaterials.

Materials	BET SSA (m^2^/g)	Materials	BET SSA (m^2^/g)
ACT1	382	Ni/ACTU1	263
ACT2	367	Ni/ACTU2	277
Ni/ACT1	233	NiW/ACTU1	214
Ni/ACT2	225	NiW/ACTU2	233
NiW/ACT1	218	NiWAu/ACTU1	290
NiW/ACT2	179	NiWAu/ACTU2	280
NiWAu/ACT1	220		
NiWAu/ACT2	215		

**Table 3:** Au particle size from XRD and TEM data.

Materials	Average Au Particle Size by DRX (nm)	Average Au Particle Size by TEM (nm)
NiWAu/ACT1	4.2	5.4
NiWAu/ACT2	4.0	6.3
NiWAu/ACTU1	3.8	4.8
NiWAu/ACTU2	5.4	5.5

**Table 4:** EDX quantitative analysis of trimetallic nanomaterials NiWAu NPs supported on Al_2_O_3_-CeO_2_-TiO_2._

NiWAu/ACT1		NiWAu/ACTU1	
Chemical Elements	ms%	Chemical Elements	ms%
O	45.8	O	44.3
Al	36.6	Al	43.5
Ti	7.0	Ti	5.3
Ni	5.0	Ni	1.1
Ce	1.0	Ce	2.2
W	2.3	W	2.2
Au	2.3	Au	1.4
Total	100	Total	100

**Table 5:** Metal surfaces (**S_G_**) and densities of metals.

Metal	S_G_ (m^2/^/g)	ρ (g/cm^3^)
Ni	654	8.90
W	753	19.35
Au	266	19.32

**Table 6:** Total dispersion (%) and metal crystallite sizes of the NiWAu/ACT nanomaterials by TPD-H_2_.

Sample	BET Area (m^2^/g)	Average Au Particle Size (nm) ^a^	HTC ((μmol H_2_/gcat)	TPD-H_2_ (H/M = 1 μmol H_2_/gcat)	% D (H/M)	MCS (nm) ^b^
NiW/ACT1	214	-	0.85	0.60	70	1.5
NiW/ACT2	179	-	0.85	0.63	74	1.4
NiW/ACTU1	218	-	2.47	0.65	26	3.3
NiW/ACTU2	179	-	2.47	0.55	22	3.9
NiWAu/ACT1	220	4.2	0.99	0.46	46	1.8
NiWAu/ACT2	215	4.0	0.99	0.50	50	1.7
NiWAu/ACTU1	290	3.8	0.99	0.33	33	2.6
NiWAu/ACTU2	280	5.4	0.99	0.41	41	2.1

^a^ Calculated from XRD data, with Scherrer’s equation. ^b^ MCS (nm) calculated from TPD-H_2_ data. MCS = average metal crystallite size. % D = percentage of metallic dispersion.

## Data Availability

Data are contained within the article.
